# Professional Gossip

**Published:** 1849-04

**Authors:** 


### PROFESSIONAL GOSSIP.

My partner, Dr. B., suggested the plan of making ilets for whole-sett springs out of half-round wire, and using a hemispherical-headed pin, (instead of a screw,) solder fast to the stiles, bend the half-round wire around the pin, and push both ends together into the springs. We have tried them in two whole-setts since, and find them to work exceedingly well.

A gentleman, farmer in Saline county, Mo., had the two central incisors of his wife's upper denture extracted; procured two plate teeth, and went to work to "put in teeth" for his wife. He took an impression with Fuller's earth, made a cast of beeswax! and with a hatchet, a shoemaker's pinchers, an awl, and a large prairie plow-file, plated out a half-dollar, mounted the teeth, turning up parts of the teeth for backs, and curving others for clasps; he needed no blow-pipe nor solder; he finished the job, and the lady wore it. *I saw it.*

A NEGRO boy with a hare-lip may be seen on the steamboat Sacramento—Capt. A. Huson.

A FELLOW practising dental surgery in Missouri, tells me has inserted pivot teeth where the fang had been extracted! and says the bone *drills quite easy!*

THERE is a dentist, in Lafayette, Ia., who *says* he left Pittsburgh because his practice was too heavy for him; has extracted more than a barrel of teeth; refuses to subscribe for either of the dental periodicals, because he has read it all in the books long ago; has Snell and Fitch!! X. E. X

